# Supplementary material for: Deep learning via LSTM models for COVID-19 infection forecasting in India
Source: PLoS One. 2022 Jan 28;17(1):e0262708. doi: 10.1371/journal.pone.0262708 (PMC8797257; doi:10.1371/journal.pone.0262708)
Supplement: Attachment — Submitted filename: PLOS_One_COVID19IndiaReview.pdf [file pone.0262708.s002.pdf]

Dear Editorial team

The authors sincerely thank the Editor and Reviewers. We have prepared a new version with changes in results and presentation to address the review comments. Please note that the updates are shown in blue in the paper along with our response here.

We made major changes with the following:

1. All visualisation and prediction results have been updated using data till 27th September 2021 to cover the second wave of infections in India.
2. The results and discussions have been updated to reflect the updated predictions.
3. Typos and grammatical issues have been fixed.

Reviewer #1: In the presented form, the authors use three LSTM models to forecast the situation of COVID-19 cases in different cities of India. Only LSTM models are used without any justification why other forecasting models available in theory would fail. This research does not seem to build models out of data, rather an application-based work is presented. The purpose seems to figure out whether LSTM model would succeed in modelling covid-19 data in India. In that case, the authors succeeded. The authors thank the reviewer for these positive comments.

Thus, comparative work is limited in scope. Though lot of work has been presented in literature on covid-19 forecasting (and quoted as well in literature review), but authors fail to simply identify why some prominent ones would have failed on cases from Indian cities. In our revision, we have highlighted in Section 2.1 which forecasting methods have failed when compared to actual cases. This has been highlighted in blue in the paper. The goal of the paper is to apply novel deep learning methods and identify which type of LSTM model performs the best.

Minor corrections needed:

1. Grammar needs to be addressed as there are some mistakes in the document.

We have fixed grammatical issues in the revision.

2. The description after equation (1) does not define ‘g” used in equation (1). We revised this by denoting g as c, which refers to the memory cell.

3. The paragraph before Figure 3 states that input sequence is x(t) and output sequence is y(t), but in Figure 3, output sequence y(t) is not stated. Please explain. We have updated the caption of Figure 3 to explain this further.

4. At the end of section 1, authors state that section 3 presents methodology with data analysis, but section three presents proposed networks only, without data analysis. It is also stated at the end of section 1, that Section 4 presents experiments and results, whereas we only find data collection in the form of Tables and Figures. Please rephrase this paragraph.

We updated Section 3 where we include data analysis and now the last paragraph in Section 1 reads well.

The authors sincerely thank the reviewer for their valuable comments.

Reviewer #2: This manuscript by Chandra et al., aims to develop deep learning models including recurrent neural networks, in particular long short term memory(LSTMs) networks, bidirectional LSTM, and encoder-decoder LSTM models for multi-step (short-term) forecasting the spread of COVID-infections in India. The authors selected Indian states with COVID-19 hotpots and compare with states where infections have been contained and provide two months ahead forecast that shows that cases will slowly decline. According to the authors their approach is promising for long-term forecasts.

My concerns for the manuscript are:

1. The manuscript is not written scientifically. It elaborates the effects of lockdown on economy which is not pertinent to the aim of this manuscript.

The effect of lockdown on the economy is part of the literature review but not the focus of the paper.

2. "India currently (1st December, 2020) has 9,462,809 confirmed cases with 137,621 (1.45 %) deaths which makes the largest in Asia the second highest in the world after the United States. The fatality rate of COVID-19 in India is among the lowest in the world % and steadily declining. India also has one of the fastest recovery rates in the world with 429,753 (4.54 %) active cases, and ranks 8th in the world although 2nd in total cases"

This statement is not true anymore specially with the delta variant and the authors should have revised the manuscript before submission.

We have updated the above and related paragraphs and have considered cases till 27th September 2021. We updated our model with data taking into account the above comment and now the paper covers the second wave of infections.

3. I couldn't find any description or modeling for the delta variants or emerging covid variants in the manuscript.

We have included the results of the delta variant in the revised manuscript. We have used the best model and applied the data on the delta variant and given two months prediction and provided a detailed discussion of new results.

Overall I think the manuscript is not suitable for this journal.

The authors thank the reviewer for these comments. We believe that the revised paper is suitable for the journal and will provide a good contribution to the COVID-19 modelling community.

Reviewer #3: Authors used different LSTM models to forecast COVID-19 infection in India. The paper is well written and required experiments conducted to validate the results. The following comments may help to improve the paper.

The authors thank the reviewer for these comments.

Fig. 2 to Fig. 6: Text in the figures are not visible, increase the font size. We have improved the figures in the revision and upon publication, the page style will change and the figures will enlarge.

Fig. 11, sub figures (a)-(d) showed high uncertainty compared to (e) and (f), authors needs to comment about this.

This has been explained in the Discussion section with the following:

"Our long-term forecasts for two months (October and November 2021) show that there will be a steady decline in new cases in India and the respective states. We find that Delhi's two monthly forecasts provide (Figure 11) more uncertainty when compared to Maharashtra and India cases. We also notice that there is a similar level of uncertainty by LSTM and BD-LSTM models for India and Delhi datasets. The major reason the uncertainties are different when you compare Delhi and Maharashtra with the rest of India is due to the difference in the trend of cases. In Delhi, multiple peaks were observed since the first wave of infections in 2020 whereas, in the India dataset, there were only two major peaks. In Maharashtra, a minor peak was observed in November 2021 (Figure 4, Panel a) after the first wave of infections. Hence, the trends captured in the training dataset were relatively different from the states (Maharashtra and Delhi) when compared to India dataset. This suggests that the predictions for Maharashtra and Delhi are less certain since they had multiple peaks and outbreaks in the past. The second wave of infections in 2021 began in Maharashtra are currently the state that holds one of the most numbers of total cases (Table 4) and novel daily infections. "

Authors may provide more details about LSTM training. LSTM model architecture with training details has been updated in the Results section.

The authors sincerely thank the reviewer for their valuable comments.
