# Supplementary material for: Deep learning via LSTM models for COVID-19 infection forecasting in India
Source: PLoS One. 2022 Jan 28;17(1):e0262708. doi: 10.1371/journal.pone.0262708 (PMC8797257; doi:10.1371/journal.pone.0262708)
Supplement: Attachment — Submitted filename: Response Letter.docx [file pone.0262708.s001.docx]

Dear Editor

We do not have track changes since the earlier manuscript was not sent for review. It was based on Associate Editors decision that the work is not novel enough in theory, however, this is an application paper and uses latest deep learning methods to investigate the univariate and multivariate nature of time series prediction for COVID19.

Previous review comments by AE

"REASON: This paper tested three existing LSTM Deep Learning Models (LSTM, Bi-directional, and Encoder-Decoder) for forecasting COVID-19 in India. There is nothing new regarding theory in the paper. We have to reject the paper.

I am sorry that we cannot be more positive on this occasion, but hope that you appreciate the reasons for this decision."

Author response:

The above comments of the AE does not take into consideration that the paper's goal is not to contribute to theoretical developments of machine learning but to apply novel deep learning problems for modelling and forecasting. The deep learning methods are used used for univariate and multivariate prediction where spatio-temporal components are considered (i;e data from neighbouring states in India during rise of COVID19 infections). The work is novel and deserves to go through the review process.
